# Comparative study of sacroiliac screw placement guided by 3D-printed template technology and X-ray fluoroscopy

**DOI:** 10.1007/s00402-019-03207-6

**Published:** 2019-05-24

**Authors:** Wu Zhou, Tian Xia, Yi Liu, Faqi Cao, Mengfei Liu, Jing Liu, Bobin Mi, Liangcong Hu, Yuan Xiong, Guohui Liu

**Affiliations:** Department of Orthopaedics, Union Hospital, Tongji Medical College, Huazhong University of Science and Technology, Wuhan, 430022 China

**Keywords:** Three-dimensional printing, Template, Sacroiliac screw, Fluoroscopy

## Abstract

**Objective:**

To compare the clinical effect of 3D-printed template technology with X-ray fluoroscopy in assisting surgery for sacroiliac screws placement.

**Design:**

Institutional review board-approved retrospective analysis.

**Patients:**

The clinical data of 31 cases of sacroiliac complex injury between January 2015 and December 2016 were analyzed. There were 16 patients, males 11 and females 5, who underwent surgery assisted by 3D-printed template in template group, and that of contemporaneous 15 patients, males 11 and females 4, who underwent traditional surgery were gathered as fluoroscopy group. All those patients were followed up for more than 6 months.

**Main outcome measures:**

The operation time and X-ray fluoroscopy times for each screw placement, and the Matta and Majeed score were analyzed and the difference between the two group was tested.

**Results:**

All cases were followed up for 6–20 months, average 11.4 ± 0.6 months. In template group, 19 screws were implanted. Each screw spent 25–38 min, average 27.2 ± 5.3 min, and need 2–5 times fluoroscopy, average 2.7 ± 0.5. The fracture reduction quality was evaluated by Matta score scale: excellent 10, well 4, fair 2, good rate 87.5%; and pelvic function were evaluated by Majeed score scale: excellent 11, well 3, fair 2, and good rate 87.5%. In fluoroscopy group, 17 screws were implanted. Each screw spent 45–70 min, average 60.3 ± 5.8 min, and needs 11–23 times fluoroscopy, average 15.4 ± 3.5. The fracture reduction quality was evaluated by Matta score scale: excellent 7, well 6, fair 2, and good rate 86.7%; and pelvic function was evaluated by Majeed score scale: excellent 6, well 6, fair 3, and good rate 80.0%. The difference in operation time, X-ray fluoroscopy times between template group and fluoroscopy group had statistical significance. But the Matta and Majeed score had no difference between two groups.

**Conclusion:**

Compared with traditional surgery, 3D-printed template technology-assisted surgery for sacroiliac screws placement in sacroiliac complex injury patients possesses advantage such as shortened operation time and reduced X-ray exposure times. This technology improves the safety profile of this operation and should be further studied in future clinical applications.

## Introduction

Since the development of transportation and acceleration of urbanization, the sacroiliac joint complex injury caused by high-energy trauma is more and more common [1]. The sacroiliac joint, as a junction of the body and lower limb, plays a key role in maintaining the function of pelvis [2], thus the treatment after trauma is also important. Internal fixation is the main treatment for sacroiliac joint complex injury (SJI) [3], and sacroiliac screw fixation is the widely accepted one [4, 5]. However, because of the anatomic feature, there are still many problems during sacroiliac screw implantation. In traditional surgery assisted by X-ray fluoroscopy, it may injure the local vessel or nerve without real-time monitoring of the position and depth of screw [6]. On the other hand, long-time fluoroscopy leads to a lot of radiation exposure both to the patient and surgeon, which is a health hazard. The rise and rapid expansion of three-dimensional (3D) printing technology, preoperative simulation, designed template, and instruction to make sure the screw location and depth all make it much more safer for screw placement in pelvic surgery [7]. In our study, the clinical data of 28 patients with pelvic fracture and sacroiliac joint injury between January 2015 and December 2016, were retrospectively analyzed to compare the treatment effect of 3D-printed template-assisted and traditional X-ray fluoroscopy-assisted sacroiliac screw placement in treating sacroiliac joint injury.

## Materials and methods

### General information

Inclusion criterion: (1) fresh and closed pelvic fracture, (2) type Tile C1 and C2 pelvic fracture, (3) the fracture dislocation could be corrected by traction, (4) underwent sacroiliac screw fixation, (5) the clinical data were intact and follow-up time was no less than 6 months.

Exclusion criterion: (1) old or open pelvic fracture, (2) severe lumbosacral trunk nerve injury, (3) non-screw internal fixation, (4) the clinical data were incomplete, or follow-up time was less than 6 months.

There were altogether 31 pelvic fracture patients brought into this study (Table 1), male 23 and female 8, age from 18 to 68-year-old (average 47). All patients signed informed consent, and the study was approved by the ethics committee of Tongji Medical College, Huazhong University of Science and Technology, the register number was 2015-S368. There were 16 patients, males 11 and females 5, who underwent surgery assisted by 3D printing template recruited as template group, age from 18 to 68 (average 47.2), 13 traffic trauma and 3 fall injury. According to Tile classification, there were 13 type C1 and 3 type C2. About associated injury, there were two hemorrhagic shocks, two urethral injuries, five pubic symphysis separations, five fractures of unilateral pubic rami, and five fractures of bilateral pubic rami. There were six cases with symptom of numbness of lower limb post trauma, and the symptom relieves a lot after traction. The patients got operation 9–13 days (average 10.9) after injury. There were contemporaneous 15 patients, males 11 and females 4, who underwent traditional surgery recruited as fluoroscopy group, aged 18–65 (average 47.1), 13 traffic traumas and 2 fall injuries. According to Tile classification, there were 13 type C1 and 2 type C2. About associated injury, there were two hemorrhagic shocks, two urethral injuries, six pubic symphysis separations, six fractures of unilateral pubic rami, and five fractures of bilateral pubic rami. There were nine cases with symptom of numbness of lower limb post trauma, and the symptom relieves a lot after traction. The patients got operation 8–13 days (average 9.7) after injury. All those patients were examined to be positive for pelvic compression and separation test and followed up no less than 6 months.

**Table 1:** The general information of two groups

Characteristics	Template group	Fluoroscopy group	Value of *t* or *χ*^2^	*P* values
Case number	16	15	–	–
Gender				
Male	11	11	0.079	> 0.05
Female	5	4		
Mean age	47.2 ± 0.8	47.1 ± 0.5	0.414	> 0.05
Cause of injury				
Traffic injury	13	13	0.168	> 0.05
Falling injury	3	2		
Tile typing				
C1	13	13	0.168	> 0.05
C2	3	2		
Associated injury				
Haemorrhagic shock	2	2	0.330	> 0.05
Urethral injury	2	2		
Symphysiolysis	5	6		
Unilateral pubic rami fracture	5	6		
Bilateral pubic rami fracture	5	5		
Numbness of lower limb	6	9		
Mean time interval from injury	10.9 ± 2.1	9.7 ± 1.8	1.703	> 0.05

### Treatment process

#### Pre-operation preparation

The patients were given supportive treatment such as vital sign monitoring and fluid infusion. The CT scanning and 3D reconstruction were to be obtained once the patient condition was stable, and the slice thickness was limited to be 0.5 mm to assess the fracture exactly. Supracondylar traction of femur was necessary until operation when there was an iliac fracture combined with obvious vertical dislocation, and the traction weight was 1/10 to the body weight (6–8 kg). The pre-operation time sustained to 8–13 days. In template group, the CT data were saved as DICOM format in mobile hard disk, and imported to a computer for further disposition. With the help of software Mimics 14.0 (Materialise corporation, Belgium), the 3D pelvic model was easy to be reconstructed, and the surgeon could simulate pelvic fracture reduction. On the basis of injury side ilium after reduction, the sacroiliac screw template was designed and printed, meanwhile the screw diameter and length were also confirmed. The process of template designation was as follows [8].

The DICOM data were imported to Mimics, to reconstruct 3D pelvic model. Draw two lines: one to pass posterior and anterior superior iliac spine, another along with the longitudinal axis of femur. Choose the crossing point as initial point, the central point of terminal plate of sacral vertebrae 1 as end point. The distance between the two points is the screw channel. Simulate nailing process, observe and measure the breadth in three-dimensional form to make sure of the length and diameter of screw. When screw channel is confirmed, the sleeve of template could be designed according to its axis, with a 2-mm inner diameter and a 10-mm outer diameter. On the other hand, the anatomical character of local iliac crest is collected, and based on which an anastrophic basic template is established. Then combine the screw sleeve and basic template to produce template rudiment. Get through the sleeve channel, add two 1.5-mm pilot holes on both sides of the sleeve, and the individualized sacroiliac screw template is ready.

#### Operation techniques

The surgical procedure was conducted by the same team who had rich experience in pelvic surgery. Prone position was recommended after general anesthesia. Pelvic anterior–posterior and lateral position, inlet and outlet view were observed by fluoroscopy, to evaluate the fracture position. When vertical displacement persisted, large dose of traction of the ipsilateral lower limb was performed to reduce the dislocation. In template group, in accordance with pre-operation plan, a 4 cm curved incision along the iliac crest was made to expose segmental posterior superior iliac spine and proximal iliac crest. Soft tissue on the outer table iliac was peeled off, the template was attached onto outer table iliac, which was fastened temporarily by two 1.5-mm Kirschner wires (K-wire). Along with the sleeve, place a 2.0-mm K-wire into the screw channel as guiding wire. Remove the template and insert 7.3-mm cannulated screw (Beijing LiBeiEr Bioengineering Institute Limited Company). In fluoroscopy group, in accordance with traditional experience, the guiding K-wire was placed through the mid-lower third intersection of connecting line of posterior and anterior superior iliac spine. Then by means of repetitious X-ray fluoroscopy, adjust the orientation and depth step by step. After confirmation under K-wire, measure the length and insert suitable cannulated screw. In both groups, inlet and outlet view were observed by fluoroscopy once more when the operation was complete.

#### Postoperative management

Routinely preventive antibiotic application should sustain 48 h post operation. Both X-ray and CT scanning and 3D reconstruction of pelvic anterior–posterior and lateral position were captured again on the day after operation. The exercise of lumbodorsal muscles and lower limb muscles strength was also carried out the first day after operation. The patients were told to come to the hospital and take pelvic X-ray at 1 month, 3 months, 6 months, and 12 months after operation, and the time for weight bearing depended on the fracture union status.

### Observational index and evaluation criterion

Observe and record the time for inserting single screw, fluoroscopy times for each screw and follow-up time. Fracture reduction effectiveness was evaluated by Matta scale [9]: excellent (< 4 mm displacement), good (4–10 mm displacement), fair (10–20 mm displacement), and poor (> 20 mm displacement). The pelvic function outcome was assessed at the last follow-up by Majeed score [10]: excellent (> 85), good (70–84), fair (55–69), and poor (< 55). The complication was also observed and recorded.

### Statistical analysis

All data were assessed by software SPSS (version 13.0, SPSS, American). Measurement data are shown as ${\bar{\text{x}}} \pm {\text{SD}}$, and compared by *t* test for difference between the two groups. Enumeration data are showed as ratio, and compared by *χ*^2^ test for difference between the two groups. *P* < 0.05 was regarded as statistically difference.

## Results

All patients were followed up from 6 to 20 months, template group 6 to 19 months with average 10.9 ± 0.6, and fluoroscopy group 6 to 20 months with average 11.1 ± 1.5. There was no statistical difference in follow-up time (*t* = 0.493, *P* > 0.05). The screw number was 19 for template group and 17 for fluoroscopy group. The time for each screw insertion was (27.2 ± 5.3) min in template group, and (60.3 ± 5.8) min in fluoroscopy group, and there was statistical difference between two groups (*t* = 16.603, *P* < 0.01). The fluoroscopy times for each screw were (2.7 ± 0.5) in template group, and (15.4 ± 3.5) in fluoroscopy group, and there was statistical difference between two groups (*t* = 14.375, *P* < 0.01) (Table 2).

**Table 2:** Clinical results of two groups

Characteristics	Total crew numbers	Mean time for each screw	Mean times for fluoroscopy	Satisfactory rate of Matta score	Satisfactory rate of Majeed score
Template group	19	27.2 ± 5.3	2.7 ± 0.5	14/16	14/16
Fluoroscopy group	17	60.3 ± 5.8	15.4 ± 3.5	13/15	12/15
*t* values	–	16.603	14.375	–	–
*χ*^2^ values	–	–	–	0.005	0.322
*P* values	–	< 0.01	< 0.01	> 0.05	> 0.05

**Fig. 1:**
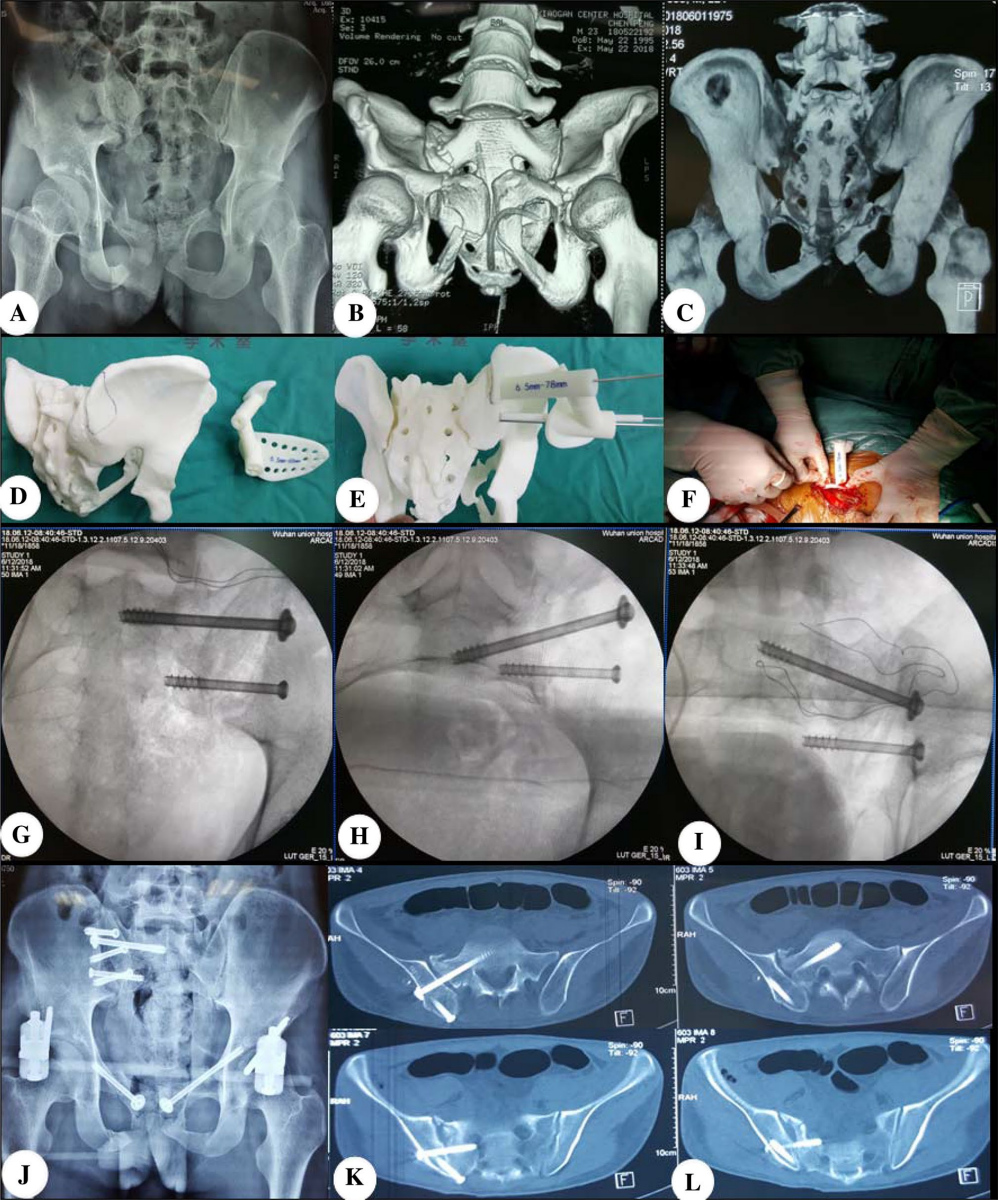
A patient in template group was presented. **a** Pelvic anterior posterior position X-ray showed bilateral pubic ramus fracture, **b**, **c** CT scanning and 3D reconstruction showed the right sacroiliac joint and ilium fracture, indicating a Tile’s C2 fracture, **d**, **e** design and print 3D pelvic model and template pre-operation, **f** place the template and insert Kirschner wire as guiding pin, **g**–**i** inlet and outlet view and anterior–posterior position of pelvic was observed by fluoroscopy, **j**–**l** the pelvic X-ray and CT showed the two sacroiliac screws in good position and good length

Assessed by Matta scale, the excellent and good rate of fracture reduction was 87.5% (14/16) in template group and 86.7% (13/15) in fluoroscopy group, and there was no statistical difference (*χ*^2^ = 0.005, *P* > 0.05). Assessed by Majeed scale, the excellent and good rate of pelvic function was 87.5% (14/16) in template group and 80.0% (12/15) in fluoroscopy group, and there was no statistical difference (*χ*^2^ = 0.322, *P* > 0.05). Typical case as in Fig. 1.

The reduction of fracture and bone union was satisfying in both group, and there was no internal fixation loosening or fracture reduction lost. In template group, the numbness of perineal exacerbated in two patients with apparent vertical displacement, and relieved after given neurotrophic drug treatment 3.5 months later. In fluoroscopy group, the numbness of perineal exacerbated and combined with gatism in one patient, and relieved after removed of screw the after day and given neurotrophic drug treatment 4.2 months later. There were still three other patients presenting transient worsening of neurological symptoms.

## Discussion

Nowadays, in orthopeadic field, it was common to see that 3D-printed model of bone fracture, reverse template, individualized implants and so on were used in clinical practice [11–13]. In this study, we designed and produced iliac-adhered guiding plate, to assist the insertion of sacral–iliac screw, and got idea therapeutic effect.

### The advantage of 3D-printed template in assisting sacral–iliac screw insertion

There are four advantages for 3D-printed template in assisting sacral–iliac screw insertion compared with traditional X-ray-assisted surgery as follows: (1) improving the accuracy and security in screw insertion (2) shortening the time for surgery procedure (3) decreasing the radiation exposure for both doctor and patient (4) enhancing the communication between doctor and patient. The sacroiliac joint complex is featured with complicated anatomy and surrounded by dense vessels and nerves [2, 14]. So it is dangerous to insert sacral–iliac screw percutaneous with the risk of vessel and nerve injury, which makes it a difficulty and challenge for the surgeon. Traditionally, X-ray fluoroscopy-assisted surgery is not so safe for its poor accuracy and non-real-time monitoring. So to ensure the safety of surgery, the surgeon usually resorts to long-term and reduplicated fluoroscopy to enhance the accuracy of screw insertion; while, with the help of 3D printing technology, the surgeon could analyze the fracture character visually and experience virtually, the operation procedure to make sure of the efficacy of operation.

In our retrospective study, each screw insertion in fluoroscopy group spent an average of 60 min, and needed an average of 15 times fluoroscopy, which was similar to other scholars [15, 16]. However, in template group, each screw insertion just spent average 28 min and needed average 3 times fluoroscopy, which was similar to the results of Yang et al. [17]. The operation and fluoroscopy times were reduced a lot compared with fluoroscopy group. Meanwhile, the radiation exposure for both doctor and patient decreased a lot too. On the other hand, in the procedure of template production, we could manipulate the electronic model to simulate the reduction of fracture and define the entry point, direction, and length of screw, to ensure the security and shorten the operation time. And by presenting a 3D-printed model of equal proportion, the doctor could explain the illness, operation difficulty and possible perioperative risk much better to the patient [18], which could improve the communication efficiency between doctor and patient and then improve the satisfaction degree and compliance of the patient.

### The indication of 3D printing technology in assisting sacral–iliac screw insertion

Although has its advantage in assisting surgery obviously, but 3D printing technology also has its limitation. Zhang et al. [15] pointed out that percutaneous screw insertation was only suitable for cases without obvious dislocated fractures, or could be reducted by traction. In our study, the patients recruited were all consistent with the above standard. So we consider that sacroiliac joint complex injury patient with irreducible fracture or osteoporosis as not appropriate for sacroiliac screw fixation.

### The essentials of operation directed by 3D-printed template and its announcements

In our experience, the pelvic anterior–posterior and lateral position, inlet and outlet view should be observed by fluoroscopy before operation to assess the reduction effect of traction. When the operation begins, try to reduct the fracture by pry-poking or reverse-rotation via Schanz screw. Open reduction internal fixation is the alternative scheme in case of poor reduction. For another thing, the 3D-printed template needs to rely on the anatomic landmark of iliac bone, so it is important to peel off the attached soft tissue thoroughly on the iliac crest; and in this process, it may injure the local soft tissue and damage the bone blood supply for fully stripping, or cause a deviation and decrease the security of operation for deficiently stripping. In our study, two cases were excluded for the changed operation plan for poor reduction, and another two cases showed a transient worsening of the neurological symptoms, which may be due to the severe displacement and nerve drag injury after reduction, or the nerve irritability during the screw insertion. Yang et al. [17] also pointed out that open reduction was necessary when closed reduction was unsuccessful.

There are still several shortcomings about the study as follows: it is a retrospective study; the sample size is not big enough; there is systematic error for analyzing the short-term and long-term complications. Furthermore, the application of 3D printing technology in clinical practice is still at its early stage, and how to improve the fracture reduction skill, and how to enhance the template accuracy both need to be further studied. At last, the comparison between 3D printing technology and 3D navigation technology also needs to be studied.

In conclusion, 3D-printed template technology-assisted surgery for sacroiliac screws placement in sacroiliac complex injury patients possesses advantage such as shortened operation time and reduced X-ray exposure times. This technology improves the safety profile of this operation and should be further studied in future clinical applications.

## References

[1] Coccolini F, Stahel PF, Montori G (2017). Pelvic trauma: WSES classification and guidelines. World J Emerg Surg.

[2] Soisson O, Lube J, Germano A (2015). Pelvic belt effects on pelvic morphometry, muscle activity and body balance in patients with sacroiliac joint dysfunction. PLoS One.

[3] Wu T, Chen W, Li X (2015). Biomechanical comparison of three types of internal fixation in a type C zone II pelvic fracture model. Int J Clin Exp Med.

[4] Elzohairy MM, Salama AM (2017). Open reduction internal fixation versus percutaneous iliosacral screw fixation for unstable posterior pelvic ring disruptions. Orthop Traumatol Surg Res.

[5] Bousbaa H, Ouahidi M, Louaste J (2017). Percutaneous iliosacral screw fixation in unstable pelvic fractures. Pan Afr Med J.

[6] Eastman JG, Routt MLC (2015). Correlating preoperative imaging with intraoperative fluoroscopy in iliosacral screw placement. J Orthop Traumatol.

[7] Chen X, Xuanhuang C, Guodong Z (2017). Accurate fixation of plates and screws for the treatment of acetabular fractures using 3D-printed guiding templates: an experimental study. Injury.

[8] Zhang Y, Wen L, Zhang J (2017). Three-dimensional printing and computer navigation assisted hemipelvectomy for en bloc resection of osteochondroma. Medicine.

[9] Matta JM, Tornetta P (1996). Internal fixation of unstable pelvic ring injuries. Clin Orthop Relat Res.

[10] Majeed SA (1989). Grading the outcome of pelvic fractures. J Bone Jt Surg Br.

[11] Zhang Y, Zhang L, Sun R (2018). A new 3D printed titanium metal trabecular bone reconstruction system for early osteonecrosis of the femoral head. Medicine.

[12] Zheng P, Yao Q, Xu P (2017). Application of computer-aided design and 3D-printed navigation template in Locking Compression Pediatric Hip Plate ^**TM**^ placement for pediatric hip disease. Int J Comput Assist Radiol Surg.

[13] Guo F, Dai J, Zhang J (2017). Individualized 3D printing navigation template for pedicle screw fixation in upper cervical spine. PLoS One.

[14] Amir Abdul-Jabbar M, Emre Yilmaz M, Joe Iwanaga DP (2018). Neurovascular relationships of the S2AI screw placement: an anatomical study. World Neurosurg.

[15] Zhang R, Yin Y, Li S (2018). Percutaneous sacroiliac screw versus anterior plating for sacroiliac joint disruption: a retrospective cohort study. Int J Surg.

[16] Ecker TM, Jost J, Cullmann JL (2017). Percutaneous screw fixation of the iliosacral joint: a case-based preoperative planning approach reduces operating time and radiation exposure. Injury.

[17] Yang H, Lei Q, Cai L (2018). Treatment of unstable pelvic fractures by cannulated screw internal fixation with the assistance of three-dimensional printing insertion template. Zhongguo Xiu Fu Chong Jian Wai Ke Za Zhi.

[18] Li FN, Huang X, Wang K (2018). Preparation and assessment of an individualized navigation template for lower cervical anterior transpedicular screw insertion using a three-dimensional printing technique. Spine.

